# Percutaneous transhepatic digital single-operator cholangioscopy-guided laser lithotripsy in treating difficult intrahepatic duct stone in surgically altered anatomy

**DOI:** 10.1055/a-2164-0619

**Published:** 2023-09-27

**Authors:** Alan Chuncharunee, Kesinee Yingcharoen, Manus Rugivarodom, Varayu Prachayakul

**Affiliations:** 1 Siriraj GI Endoscopy Center, Division of Gastroenterology, Department of Internal Medicine, Faculty of Medicine, Siriraj hospital, Mahidol University, Bangkok, Thailand; 2 Division of Gastroenterology and Hepatology, Department of Medicine, Ramathibodi Hospital, Mahidol University, Bangkok, Thailand

Endoscopic intrahepatic duct (IHD) stone management in patients with surgically altered anatomy is a major technical challenge 1 2 .

A 74-year-old man with a history of hilar resection with Roux-en-Y hepaticojejunostomy from a hilar tumor presented with a history of cholangitis. Magnetic resonance imaging showed multiple large left IHD stones ( Fig. 1 ). Despite balloon enteroscopy-assisted endoscopic retrograde cholangiopancreatography and percutaneous cholangioplasty, residual impacted IHD stones were present within the left IHD proximal to the stricture ( Fig. 2 ). After multidisciplinary team discussion, the patient underwent percutaneous transhepatic cholangioscopy-guided laser lithotripsy ( Video 1 ).

**Fig. 1:**
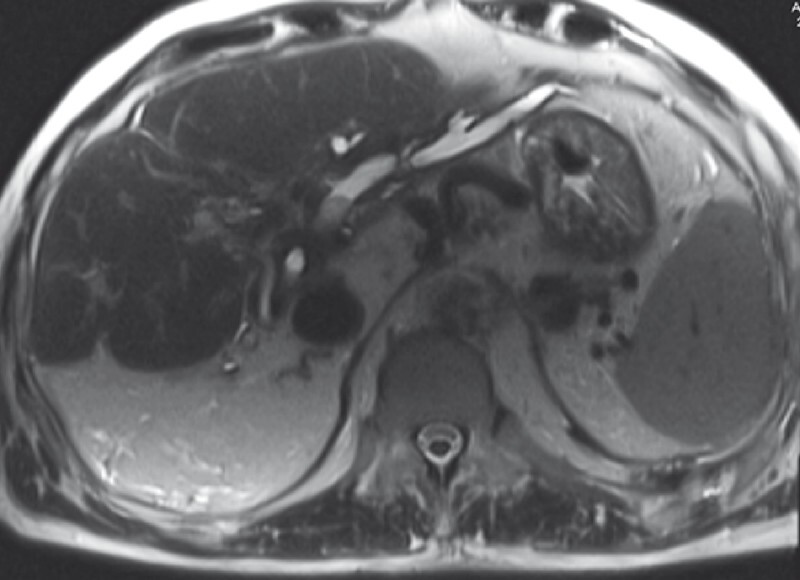
Magnetic resonance imaging showed multiple large and impacted left intrahepatic duct stones.

**Fig. 2:**
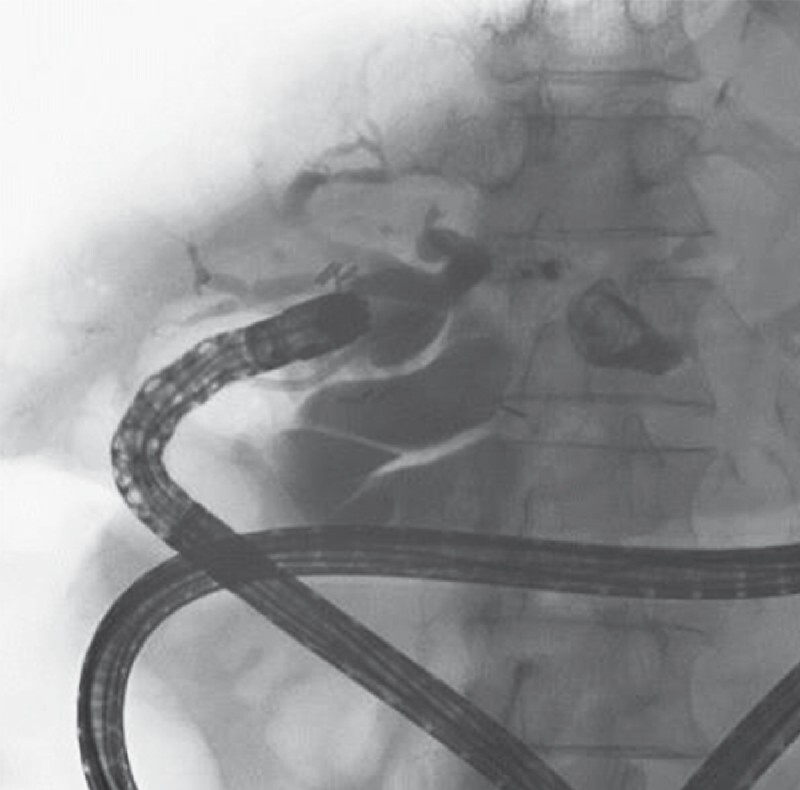
Cholangiogram showed residual intrahepatic duct stone proximal to the intrahepatic duct stricture.

**Video 1** Presentation of the case and percutaneous transhepatic cholangioscopy-guided laser lithotripsy.


The percutaneous tract was dilated up to 14 Fr and left to mature for 8 weeks. General anesthesia and prophylactic antibiotic administration were performed. The guidewire (0.035-inch, Visiglide; Olympus, Tokyo, Japan) via the percutaneous transhepatic biliary drainage catheter (14 Fr, biliary catheter; Cook Medical, Bloomington, Indiana, USA) was inserted into the left IHD to reach the jejunum beyond the anastomosis. The catheter was then removed. A novel short-length (65 cm) cholangioscope (SpyGlass Discover Digital Catheter; Boston Scientific, Marlborough, Massachusetts, USA) was introduced over the wire into the IHD ( Fig. 3 ). The previously placed stent was removed using a SpySnare (Boston Scientific). A laser fiber (Jena Surgical, Brüsseler, Germany) was introduced. We performed laser lithotripsy from the central portion and moved upstream to the periphery. Stone fragments were flushed downstream into the jejunum until completely cleared. The patient recovered well and was discharged on Day 2 after the procedure.

**Fig. 3:**
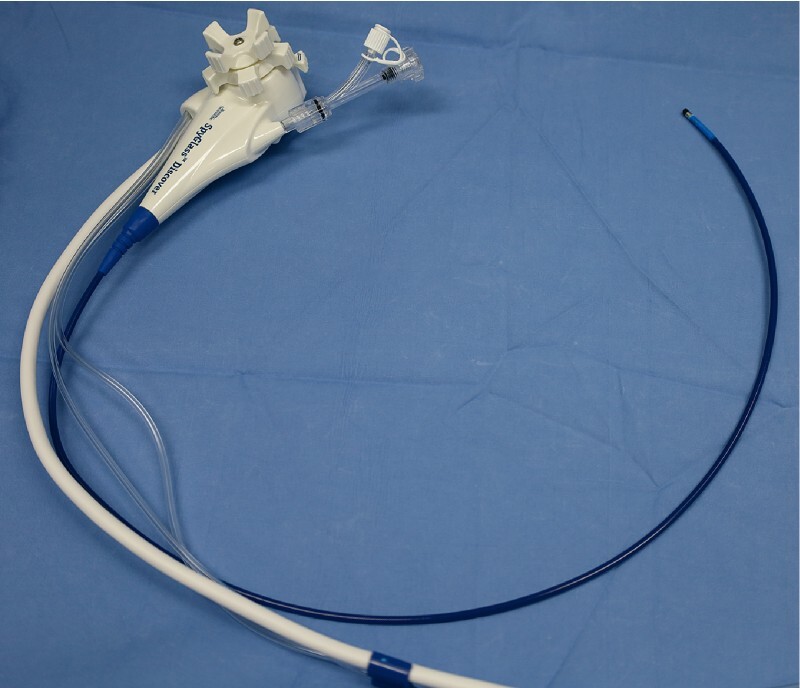
A novel short-length (65 cm) cholangioscope.

For technical consideration, the percutaneous access tract of at least 12 Fr in size should be left for at least 4–8 weeks to ensure tract maturation. The short-length cholangioscope was preferable for percutaneous access owing to the ease of scope maneuverability and high scope stability, as well as low risk of endoscopy-related infection.

Endoscopy_UCTN_Code_TTT_1AR_2AH

## Notes

Endoscopy E-Videos

https://eref.thieme.de/e-videos
*E-Videos* is an open access online section of the journal *Endoscopy* , reporting on interesting cases and new techniques in gastroenterological endoscopy. All papers include a high-quality video and are published with a Creative Commons CC-BY license. Endoscopy E-Videos qualify for HINARI discounts and waivers and eligibility is automatically checked during the submission process. We grant 100% waivers to articles whose corresponding authors are based in Group A countries and 50% waivers to those who are based in Group B countries as classified by Research4Life (see: https://www.research4life.org/access/eligibility/ ). This section has its own submission website at https://mc.manuscriptcentral.com/e-videos
